# Fossils Shed a New Light on the Diversity and Disparity of the Family Limoniidae (Diptera, Nematocera)

**DOI:** 10.3390/insects12030206

**Published:** 2021-03-01

**Authors:** Wiesław Krzemiński, Iwona Kania-Kłosok, Ewa Krzemińska, Jan Ševčík, Agnieszka Soszyńska-Maj

**Affiliations:** 1 Institute of Systematics and Evolution of Animals, Polish Academy of Sciences, Sławkowska 17, 31-016 Kraków, Poland; wieslawk4@gmail.com (W.K.); ekrzeminska@poczta.fm (E.K.); 2 Department of Biotechnology, Institute of Biology and Biotechnology, University of Rzeszów, Zelwerowicza 4, 35-601 Rzeszów, Poland; 3 Department of Biology and Ecology, Faculty of Science, University of Ostrava, Chittussiho 10, 71000 Ostrava, Czech Republic; sevcikjan@hotmail.com; 4 Department of Invertebrate Zoology and Hydrobiology, Faculty of Biology and Environmental Protection, University of Łódź, Banacha 12/16, 90-237 Łódź, Poland; agasosz@biol.uni.lodz.pl

**Keywords:** fossil insects, Cretaceous, Burmese amber, new species, new genus, new subfamily

## Abstract

**Simple Summary:**

A new established subfamily (family Limoniidae) from Cretaceous Burmese amber shows a unique reduction of radial veins combined with complete set of medial veins. The discovery of this new subfamily contributes to a better understanding of the diversity and disparity of the family, and is important for further research on the evolution of this group of insects.

**Abstract:**

A new subfamily Drinosinae (Diptera, Limoniidae) is established with two fossil genera, *Drinosa* and *Decessia* gen. nov. with one new species, *Decessia podenasi* gen. et sp. nov. from Cretaceous Burmese amber. Additional description of *Drinosa prisca* is based on new material. A new subfamily shows unique reduction of radial veins combined with complete set of medial veins.

## 1. Introduction

**Figure 1:**
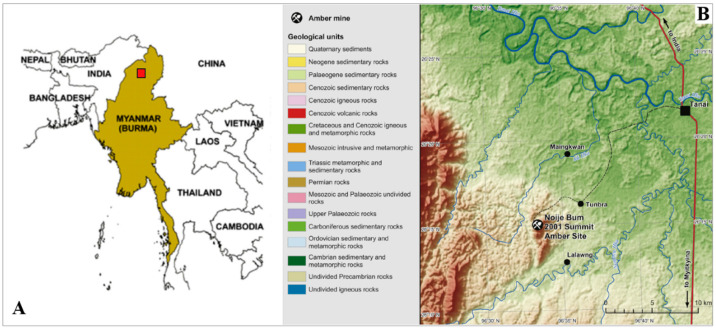
(**A**) Position of Myanmar. (**B**) Location of recent amber mining area in the Hukawng Valley, Myitkina Province, Myanmar. Compiled from data provided by known data [3,36,41,42,43], modified.

Cretaceous ambers are of great value for paleoentomological research. We find there the inclusions of descendants of the Triassic and Jurassic fauna hitherto known only from imprints in sediments, as well as the first representatives of families and genera which dominate in recent fauna [1,2,3,4,5,6,7,8]. Especially important is Burmese amber (Figure 1), aged at the beginning of the Upper Cretaceous, Cenomanian (98.79 ± 0.62 Ma [9]). This amber comprises large number of perfectly preserved complete bodies of insects which enable us to carry out research at the same scientific precision as on recent entomofauna. The most abundant in Burmese amber among insect inclusions are specimens of flies, which are often almost perfectly preserved [10]. Wing venation, antennae, legs, and especially male genitalia are the base for systematic and phylogenetic research of this group of insects, and enable us to compare fossil and recent specimens [11]. This is especially true for groups like the Limoniidae (lower Diptera, =Nematocera) with externally exposed male genitalia, which provide the necessary set of characters diagnostic to the species.

The Limoniidae are abundant in Burmese amber [12,13,14,15,16]; 16 species within 11 genera are described [10]. Diversity of this family is enormous, and in Burmese amber, along with representatives of recent genera like *Helius* Lepeletier et Serville, 1828 [17], *Gonomyia* Meigen, 1818 [18], *Dicranoptycha* Osten-Sacken, 1859 [19], also the representatives of unknown evolutionary lineages were found. Undoubtedly one of them is the genus *Drinosa* Podenas et Poinar, 2009 [13], which was not classified to any known subfamily of Limoniidae, fossil or recent because of unique set of characters.

The family Limoniidae comprises six subfamilies (Table 1), of which four: Limnophilinae Bigot, 1854 [20], Chioneinae Rondani, 1841 [21], Dactylolabinae Alexander, 1920 [22] and Limoniinae Speiser, 1909 [23] are represented in recent fauna, and two, Architipulinae Handlirsch, 1906 [24] and Eotipulinae Kalugina, 1985 [25] were dominant among the Limoniidae in the Jurassic and became extinct with the end of Mesozoic [15,26,27,28,29]. The oldest Tipulomorpha come from the Lower/Middle Triassic (ca. 245 Ma) [2,11,30,31,32], and the oldest representative of family Limoniidae is *Archilimonia youngi* Krzemiński, 1992 [27] described from the Upper Triassic of North America [27,30]. Triassic material of the Limoniidae is still fairly limited, but in many Lower Jurassic fossil localities their imprints became abundant [25,33,34,35].

In course of our study on the Mesozoic Tipulomorpha some very interesting specimens in Burmese amber were found, which represent an unknown evolutionary line of Limoniidae, thanks to which we can justify the description of the seventh subfamily of Limoniidae, Drinosinae subfam. nov., and complement an earlier discovery of a mysterious *Drinosa*.

## 2. Materials and Methods

The study was based on 10 inclusions from Burmese amber dated on 98.79 ± 0.62 Ma, (Upper Cretaceous, Cenomanian) [9] (Figure 1), two inclusions of *Decessia podenasi* gen. et sp. nov. and eight of *Drinosa prisca* [13]. The specimens were examined with the Nikon (SM Z25) stereomicroscope equipped with a Nikon digital camera (DS-Ri2), and with the Nikon SMZ 1500 stereomicroscope equipped with a Nikon DS–Fi1 camera. The measurements were taken with NIS–Elements D3.0. Drawings were completed by tracing the photographs. The wing venation nomenclature follows that of [11,39], and the terminology of male genitalia follows [40]. The measurements are given in millimeters (mm), the length of particular segments of antennae or palpus according to the pattern: antenna or palpus section number/length of this section in millimeters. The “MP” abbreviation is part of specimen traditional number from the collection of Institute of Systematics and Evolution of Animals, Polish Academy of Sciences, Kraków and it means “Muzeum Przyrodnicze” —Natural History Museum. Institutional Abbreviation: ISEA PAS—Institute of Systematics and Evolution of Animals, Polish Academy of Sciences, Kraków, Poland.

## 3. Results

### Systematic Paleontology

Order Diptera Linnaeus, 1758 [44]

Infraorder Tipulomorpha Rohdendorf, 1961 [45]

Family Limoniidae Speiser, 1909 [23]

**Figure 2:**
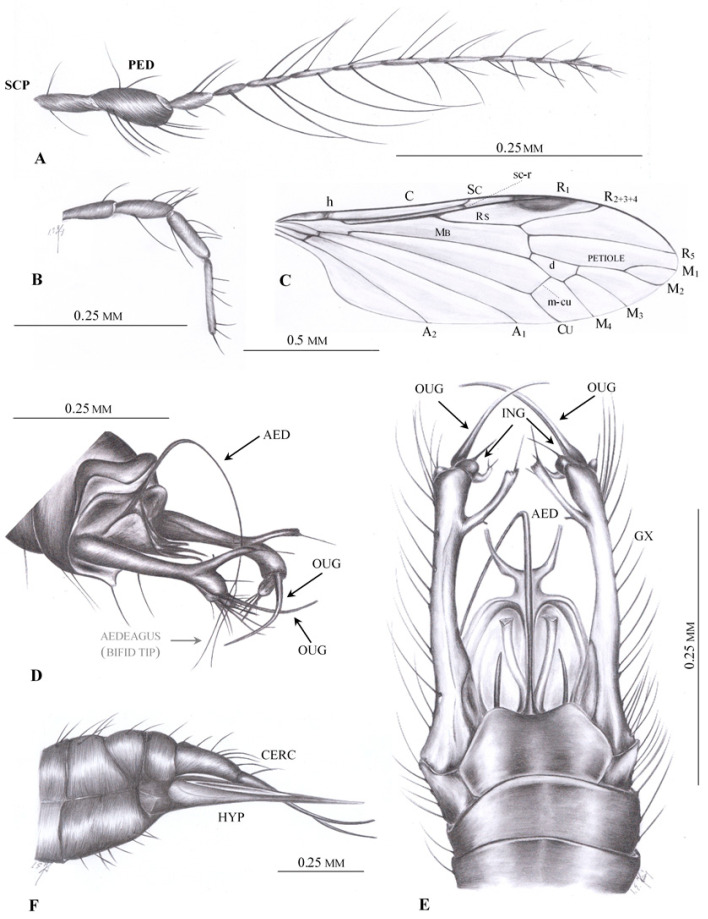
*Drinosa prisca* Podenas et Poinar, 2009 [13]. (**A**) No. MP/4068 (male) (ISEA PAS), antenna; (**B**) No. MP/4068 (male) (ISEA PAS), palpus; (**C**) No. MP/4068 (male) (ISEA PAS), wing venation; (**D**) No. MP/4068 (male) (ISEA PAS), hypopygium—lateral view, reconstruction; (**E**) No. MP/4022 (male) (ISEA PAS), hypopygium—dorsal view, reconstruction; (**F**) No. MP/4074 (female), ovipositor—lateral view. Abbreviation: scp—scape; ped—pedicel; ing—inner gonostylus; oug—outer gonostylus; gx—gonocoxite; cerc—cerci; hyp—hypogynal valvae.

**Figure 3:**
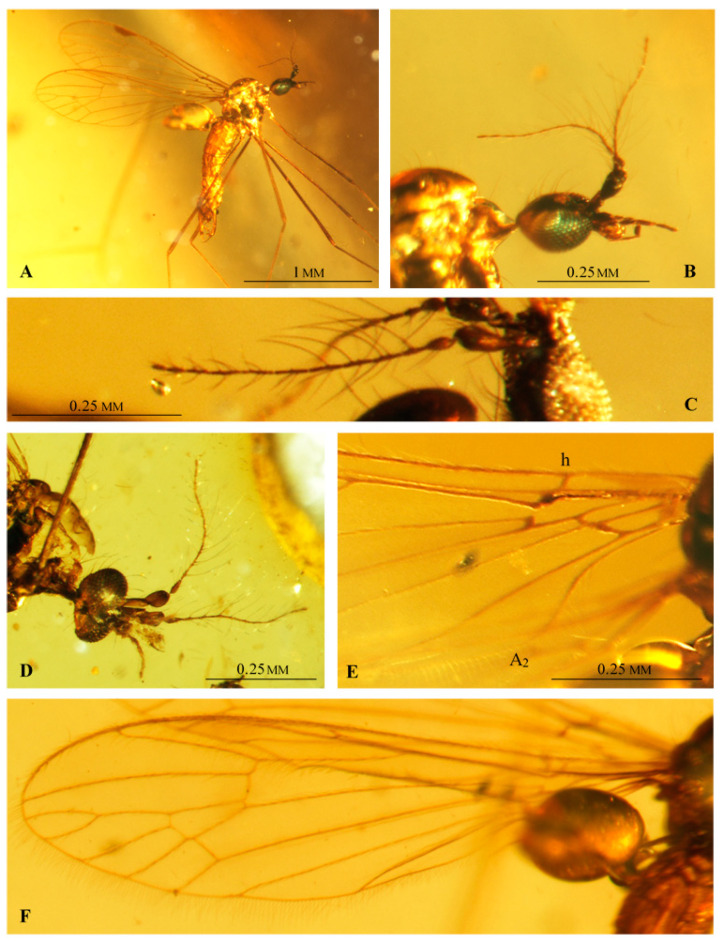
*Drinosa prisca* Podenas et Poinar, 2009 [13]. (**A**) No. MP/4022, (male) (ISEA PAS), body, lateral view; (**B**) No. MP/4022, (male) (ISEA PAS) head, lateral view; (**C**) No. MP/4068 (male) (ISEA PAS), antenna; (**D**) No. MP/4070 (male) (ISEA PAS), head, latero-ventral view; (**E**) No. MP/4068 (male) (ISEA PAS), base of wing; (**F**) No. MP/4022 (male) (ISEA PAS), wing venation.

**Figure 4:**
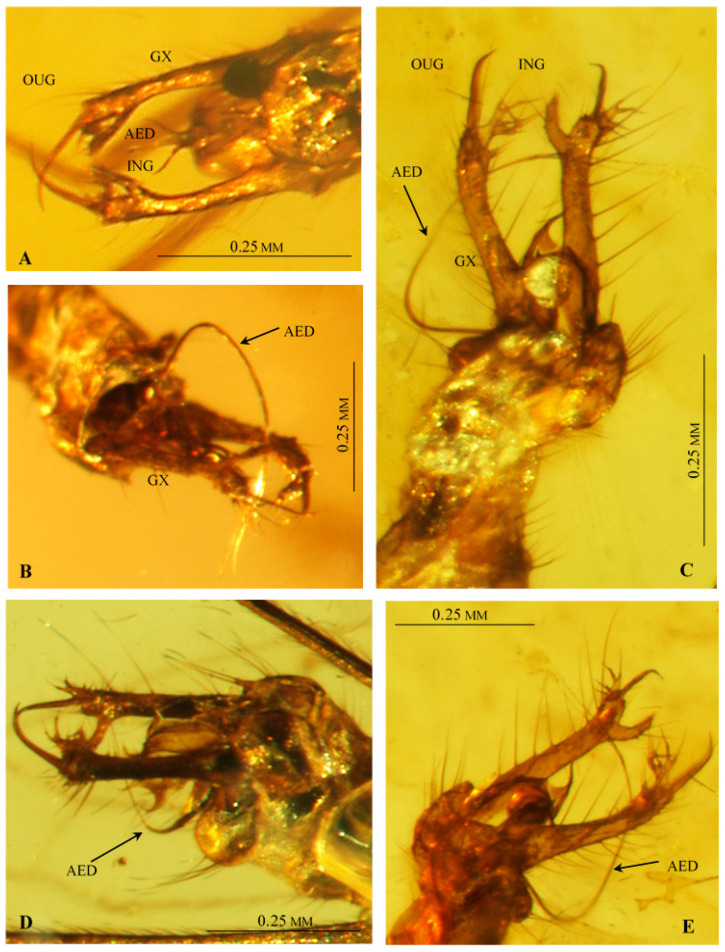
*Drinosa prisca* Podenas et Poinar, 2009 [13], hypopygium. (**A**) No. MP/4022 (ISEA PAS), dorsal view; (**B**) No. MP/4068 (ISEA PAS), latero-ventral view; (**C**) No. MP/4072 (ISEA PAS), latero-ventral view; (**D**) No. MP/4070, lateral view; (**E**) No. MP/4072 (ISEA PAS), latero-dorsal view. Abbreviation as in Figure 2.

**Figure 5:**
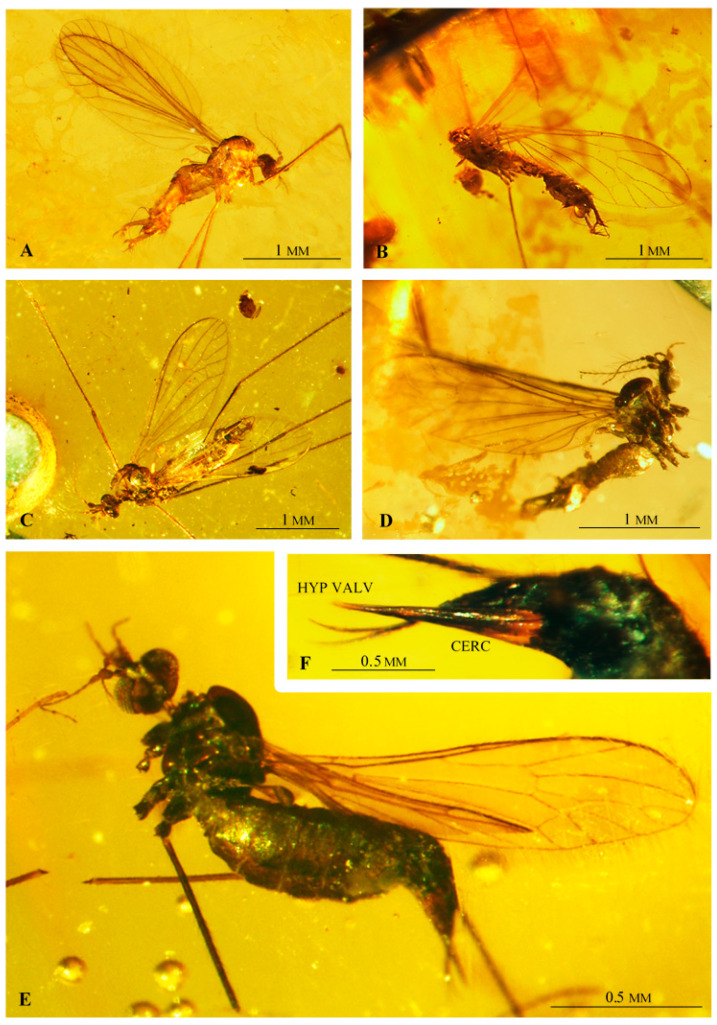
*Drinosa prisca* Podenas et Poinar, 2009 [13]. (**A**) No. MP/4072 (male) (ISEA PAS), body, lateral view; (**B**) No. MP/4073 (male) (ISEA PAS), body, lateral view; (**C**) No. MP/4070 (male) (ISEA PAS), body, latero—dorsal view; (**D**) No. MP/4068 (male) (ISEA PAS), lateral view; (**E**) No. MP/4074 (female) (ISEA PAS) body, lateral view; (**F**) No. MP/4074 (female) (ISEA PAS) ovipositor, lateral view. Abbreviation as in Figure 2.

**Figure 6:**
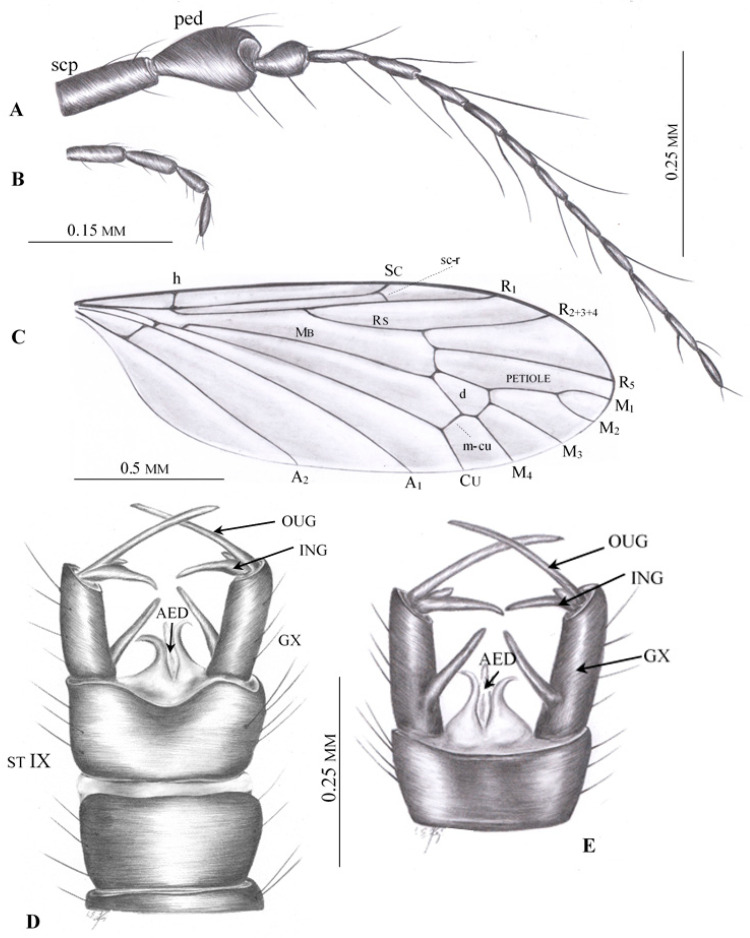
*Decessia podenasi* gen et sp. nov. No. MP/4067 holotype (male) (ISEA PAS). (**A**) antenna; (**B**) palpus; (**C**) wing venation; (**D**) hypopygium and last segments of abdomen, ventral view; (**E**) hypopygium, dorsal view. Abbreviation: scp —scape; ped—pedicel; aed—aedeagus; ing—inner gonostylus; oug—outer gonostylus; gx—gonocoxite; st IX—sternite IX.

**Figure 7:**
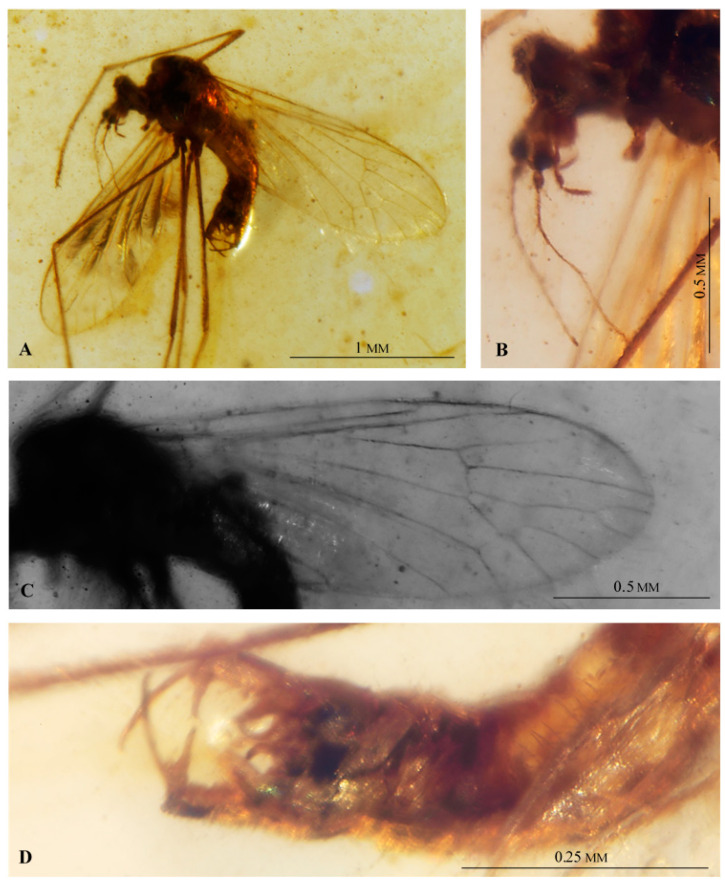
*Decessia podenasi* gen et sp. nov. No. MP/4067 holotype (male) (ISEA PAS). (**A**) body, latero-ventral view; (**B**) head, lateral view; (**C**) wing venation; (**D**) hypopygium and last segments of abdomen, ventral view.

**Drinosinae subfam. nov.**

(Figure 2, Figure 3, Figure 4, Figure 5, Figure 6 and Figure 7)

http://zoobank.org/urn:lsid:zoobank.org:act:6E1CFC71-8A4E-4029-B769-E2F12E44C84A (accessed on 28 February 2021)

**Type genus.***Drinosa* Podenas et Poinar, 2009 [13], Burmese amber, Upper Cretaceous, Cenomanian.

**Diagnosis.** Antenna: 13–14 segmented, flagellomeres long, with very long setae; 1st flagellomere expanded. Venation: Rs short, shorter than R_2+3+4_, with only two branches reaching wing margin: R_2+3+4_ and R_5_; d cell closed; four medial veins reaching wing margin: M_1_, M_2_, M_3_ and M_4_; petiole of m_1_ cell (a fused section of M_1+2_ outside the d cell; Figure 2C) present; m-cu positioned in distal part of the d cell.

**Comparison.** In contrast to all other Limoniidae, in the Drinosinae, subfam. nov., the vein Rs branches only into two veins: R_2+3+4_ and R_5_; therefore, only three radial veins are present and reach the wing margin: R_1_, R_2+3+4_ and R_5_. The crossvein r-r (R_2_) is absent. The medial field is not reduced in the Drinosinae; all four veins M_1_, M_2_, M_3_ and M_4_ are present and reach the wing margin, and the discal cell (d cell) is closed. In other subfamilies of Limoniidae except genus *Helius*, *Elephantomyia* Osten-Sacken, 1860 [19] and *Toxorhina* Loew, 1850 [46], five radial veins are present, including the crossvein r-r (R_2_).

**Description.** Body brown, ca. 2 mm long; prothorax small, scutellum rather narrow; pleura of thorax and lateral margins of mediotergite in some specimens blackish due to oxidation. Head wider than long, with medium-sized, oval eyes widely separated; vertex covered with sparse, light setae longer than scape but shorter than head. Antenna longer than head, 13–14 segmented, flagellomeres with very long verticils longer than segments bearing them; scape rather short, cylindrical or nearly cylindrical; pedicel barrel-like, longer than wide, slightly longer than scape; first flagellomere expanded medially, shorter than pedicel, other flagellomeres rather thin and oval, last flagellomeres usually shortest. Palpus short, with last palpomere longer than penultimate one; palpomeres covered by dense, erect, short setae, with additional scarce long setae, usually longer than segments bearing them.

Thorax brown, wings approximately three times as long as wide without darkened spots except dark, oval pterostigma. Wing venation: Sc ending opposite 1/2 to 2/3 of Rs; sc-r positioned close to or at tip of Sc; arculus absent, Rs almost as long as R_2+3+4_; R_1_ ending just behind half length of R_2+3+4_; cell r_2+3+4_ widely expanded in distal part, distal part of cell r_5_ very narrow; crossvein m-m present; crossvein m-cu in distal part of d-cell, rather short and straight; M_1_ and M_2_ short, petiole of m_1_ present; d cell small, trapezoidal; crossvein m-cu in 2/3 of d cell; A_1_ long, almost straight; A_2_ long, straight, approximately as long as Mb. Legs long, slender, without tibial spurs.

Male genitalia differ between the two genera and are presented in the generic descriptions.

**Remarks.** Two genera *Drinosa* and *Decessia* gen. nov. are included in subfamily Drinosinae subfam. nov.

**Genus *Drinosa*** Podenas et Poinar, 2009 [13]

2009 *Drinosa* Podenas et Poinar [13]: 471

**Type species.***Drinosa prisca* Podenas et Poinar, 2009 [13]—Burmese amber, Upper Cretaceous, Cenomanian. A monotypic genus.

**Emended diagnosis.** Antenna 13-segmented, flagellomeres long with three or four very long setae. Venation: Sc short, about half the length of wing; Rs branches into two veins reaching wing margin: R_2+3+4_ and R_5_; d cell closed; petiole of m_1_ cell is almost as long as upper margin of d cell, and approximately equal M_1_; four medial veins reach wing margin: M_1_, M_2_, M_3_, and M_4_; m-cu positioned in distal half of d cell. Male hypopygium with strongly elongated gonocoxite; two apical gonostyles are present; aedeagus is very long, thin and bifid at end, supported basally by large structure ending with two divergent appendages.

***Drinosa prisca*** Podenas et Poinar, 2009 [13]

(Figure 2, Figure 3, Figure 4 and Figure 5)

2009 *Drinosa prisca* Podenas et Poinar [13]: 473, Figure 1, Figure 2 and Figure 3 male; Figure 4 female

**Emended diagnosis.** As for the genus.

**Material examined.** MP/4022 male; MP/4068 male; MP/4069 male; MP/4070 male; MP/4071 male; MP/4072 male; MP/4073 male; MP/4074 female—Burmese amber (98.79 ± 0.62 Ma, Upper Cretaceous, Cenomanian), all housed in ISEA PAS.

**Additional description.** Antenna (Figure 2A and Figure 3B–D) longer than head, but shorter than length of head and thorax combined. Flagellomeres 2–5 with very long setae, approximately as long as three or four flagellomeres combined. Palpus (Figure 2B and Figure 3D) rather thin, palpomeres with few, not very long setae. Wing (Figure 2C and Figure 3E,F) approximately three times as long as wide; pterostigma positioned at level of d cell; Rs shorter than R_2+3+4_; origin of Rs in basal half of wing; tip of R_1_ opposite middle of d cell; d cell widened distally; arculus absent; M_1_ and M_2_ reach wing margin, cell m_1_ with petiole almost as long as upper margin of d cell and approximately equal to M_1_; four medial veins reach wing margin; crossvein m-cu at 2/3 of d cell length; both anal veins nearly straight and divergent; A_2_ terminates opposite origin of Rs.

Male hypopygium (Figure 2D,E, Figure 4A–E and Figure 5A–D) large, approximately as long as 1/4 body length (Figure 3A and Figure 5A–E), semi-inverted, ninth tergite and sternite fused. Gonocoxite greatly elongated, central part cylindrical, covered with very long setae, gonocoxite bears a long dorsomesal process at distal 1/5. Two pairs of gonostyles present, positioned apically on gonocoxite and forming together C-shaped structure; inner gonostylus very short, with two apical branches, outer gonostylus long and thin, tapering to apex and curved inwards. Aedeagus very long, thin; distal 1/4 is bifid; paramere rod-shaped, short. Additional, probably semi-membraneous structure extends between both gonocoxites, and terminates in two divergent, semicircular horns which support the basal part of an extremely long, flexible aedeagus. This structure seems less sclerotized than parameres.

Female: ovipositor (Figure 2F and Figure 5E,F) with very long, thin cerci; hypogynal valvae long, thin and stiff.

**Measurements**. Male: Body 1.60–2.28 mm long.

Head: 0.16–0.18 mm high, 0.20–0.30 mm wide. Antenna 0.45–0.60 mm long: 1/0.07–0.09 mm; 2/0.06–0.09 mm; 3/0.04–0.06 mm; 4/0.03–0.04 mm; 5/0.02–0.05 mm; 6/0.04–0.05; 7/0.04–0.06 mm; 8/ 0.04–0.05 mm; 9/0.03–0.04 mm; 10/0.02–0.04; 11/0.02–0.03 mm. Palpus 0.23–0.39 mm long (1/0.07–0.08 mm long; 2/0.04–0.08 mm long; 3/0.04–0.09 mm long; 4/0.08–0.14 mm long).

Thorax: wing 1.48–2.09 mm long, 0.35–0.67 mm wide; Rs 0.35–0.48 mm long; d cell 0.15–0.24 mm long; petiole 0.19 mm–0.24 mm; M_1_ 0.17–0.26 mm long; M_2_ 0.16–0.32 mm. Haltere 0.25–0.27 mm long.

Abdomen: hypopygium: 0.47–0.53 mm long; gonocoxite 0.28–0.29 mm long; gonostyle 0.08–0.16 mm long; aedeagus 0.47–0.81 mm long.

Female: Body 2.02 mm long. Head: antenna: 2/0.06 mm; 3/0.04 mm; 4/0.03 mm; 13/0.02 mm; palpus 0.28 mm long (1/0.03 mm long; 2/0.06 mm long; 3/0.09 mm long; 4/0.10 mm long).

Thorax: wing 1.48 mm long, 0.41 mm wide; d-cell 0.15 mm long; petiole 0.20 mm; M_1_—18 mm long; M_2_—0.15 mm long; Rs—0.30 mm long.

Abdomen: ovipositor 0.57 mm long; valvae 0.57 mm long, cerci 0.44 mm long.

**Remarks.** The ovipositor of *Drinosa prisca* was incorrectly described and illustrated in original description [13]. Authors probably studied a destroyed female specimen, with missing or invisible distal part of ovipositor. A female studied by us (no. MP/4074; Figure 2F and Figure 5F) is complete with a well visible ovipositor, which is slender, with very long both cerci and hypogynal valvae.

***Decessia* gen. nov.**

(Figure 6 and Figure 7)

http://zoobank.org/urn:lsid:zoobank.org:act:D53C4385-D7B6-4ABF-9E95-DD879AA8115C (accessed on 28 February 2021)

Type species: *Decessia podenasi* gen. et sp. nov., by present designation and monotypy. Upper Cretaceous (Cenomanian) Burmese amber.

**Etymology.** “Decessio” (Latin) = reduction; name alludes to the reduced radial field of the wing. Gender feminine.

**Diagnosis.** Antenna 14-segmented. Venation: Sc longer than half wing length; Rs branches into two veins reaching wing margin: R_2+3+4_ and R_5_; d cell closed; petiole longer than upper margin of d cell and longer than M_1_; four medial veins reach wing margin; m-cu positioned in distal half of d cell. Male hypopygium with gonocoxite not very long, with long mesobasal process half as long as gonocoxite; outer gonostylus long and thin, saber-shaped; inner gonostylus half as long as outer gonostylus, tapering to apex, with small lobe at base.

**Comparison.***Decessia* gen. nov. and *Drinosa* differ by the number of flagellomeres: antennae of *Decessia* are 14-segmented while those of *Drinosa*, 13-segmented. The two genera differ mainly by morphology of male hypopygium. In *Decessia* gen. nov. the gonocoxite is much shorter than in *Drinosa*; also position of processes on gonocoxite is different in both genera. The aedeagus in *Drinosa* is extremely long and thin, extending far beyond the hypopygium and supported by a large, additional structure; in *Decessia* the aedeagus is very short, comprised between the parameres. There are also differences in wing shape and venation: in *Decessia* gen. nov. the wing is more rounded, and the vein Sc extends beyond half wing, while the wing of *Drinosa* is more rectangular, with a well-developed anal angle, and Sc ends about mid wing; there are also differences in proportions of d cell and distal section of vein M_1_ and the petiole of m_1_, as illustrated in Figure 2C (*Drinosa*) and Figure 6C (*Decessia*).

**Description.** As for the species.

***Decessia podenasi* sp. nov.**

(Figure 6 and Figure 7)

http://zoobank.org/urn:lsid:zoobank.org:act:7AA73EA5-82AF-4206-B3A7-EC743A01E795 (accessed on 28 February 2021)

**Etymology.** The specific name is dedicated to Sigitas Podenas (Vilnius University), the eminent specialist of fossil and recent Diptera, and renowned expert on Tipuloidea.

**Diagnosis.** As for genus.

***Material examined.** Holotype MP/4067 male; paratype MP/4066 male*—Burmese amber (98.79 ± 0.62 Ma, Upper Cretaceous, Cenomanian)—housed in ISEA PAS.

**Description.** Body brown, 1.76 mm long (all measurements concern the holotype). Head (Figure 7A,B) 0.31mm high, 0.25 mm wide, with not very large, oval eyes. Antenna (Figure 6A): short, 0.58 mm long, 14-segmented: 1/0.007 mm; 2/0.09 mm; 3/0.04 mm; 14/0.03 mm, other segments of antenna approximately 0.06 mm long. Scape rather short, cylindrical; pedicel barrel-like, wide, slightly longer than scape, longer than wide; first flagellomere widened in middle part, shorter than pedicel, other flagellomeres rather tiny and oval, last flagellomere the shortest; flagellomeres 1–13 with elongate setae, much longer than width of segments bearing them and longer than length of segments where they occur. Palpus (Figure 6B and Figure 7B) 0.2 mm long, thin (1/0.04 mm long; 2/0.04 mm long; 3/0.06 mm long; 4/0.06 mm long), first palpomere elongate, as long as second one, second and third palpomeres slightly widened in distal part; last palpomere as long as penultimate one.

Thorax (Figure 7A): wing 1.80 mm long, 0.64 mm wide, approximately three as long as wide. Wing (Figure 6C and Figure 7C): vein Sc ending opposite 2/3 of vein Rs; sc-r just before before tip of Sc; arculus absent; Rs almost as long as R_2+3+4_; vein R_1_ ending just behind half the length of R_2+3+4_; cell r_2+3+4_ widely expanded distally, cell r_5_ distally narrowed; crossvein m-m present, d cell closed; crossvein m-cu straight, positioned in distal part of d-cell; M_1_ and M_2_ short, petiole 0.23 mm long, d-cell short and wide, 0.21 mm long, trapezoidal, crossvein m-cu in 2/3 length of d-cell base; A_1_ elongate, almost straight; A_2_ elongate, slightly curved at wing margin, approximately as long as Mb.

Legs (Figure 7A) slender, without tibial spurs.

Abdomen: hypopygium (Figure 6D,E and Figure 7D) 0.31 mm long; gonocoxite elongate and narrow, 0.16 mm long, with mesobasal long and thin process; outer gonostylus almost straight, c. as long as gonocoxite and twice as long as inner gonostylus; inner gonostylus tapering to the apex, tip curved; aedeagus short, barely extending beyond parameres which are massive, crescent shaped.

Female unknown.

## 4. Discussion

The Limoniidae, represented in recent fauna, are the oldest group within Tipulomorpha, and one of the oldest families of the Diptera in general [11]. Among the recent fauna, it is one of the most species-rich families, but also their fossil record is also one of the richest among the Diptera in the Mesozoic and Cenozoic. The oldest representatives of Limoniidae are known from the Upper Triassic [1,11,27,30], while the immediate ancestory lineage, the Archilimoniidae, were among the oldest known fossil flies from the Middle Triassic [11]. The first rapid radiation of Limoniidae occurred at the turn of the Triassic to Jurassic [47,48,49]. The first 70 million years of Limoniidae evolution are known only from imprints in sediments what limits our knowledge about their morphology mostly to the wing venation. Usually, male and female genitalia are only fragmentarily preserved in sediments which hinders a detailed study on morphology of these structures. Since the Lower Cretaceous the Limoniidae are documented also by inclusions in fossil resins, beginning from Lebanese amber (age 140 Ma [50,51]). The inclusions in fossil resins provide a full and certain information on morphology of entire bodies and allow comparison of fossil and recent species almost on the same level. Equipped with this knowledge we can better understand the ways of evolution and the phylogenetic relationships within family Limoniidae and all the infraorder Tipulomorpha. In this study we have decided not to perform a new phylogenetic analysis because previous analyses did not bring satisfactory results and we consider it better to wait until more similar new discoveries in the fossil record are published, to reach as broad taxon sampling as possible.

During over 210 Ma years of evolution numerous species of Limoniidae appeared and testify to the great diversity of this group in the past and today. Various evolutionary lineages (genera) show convergences in morphology, which impede tracking the processes of evolution within the family. Unfortunately, molecular research based on fossil specimens appeared unsuccessful due to breakdown of DNA during the fossilization, which concerns the sediments as well as the resins. Information contained in sensational publications proved to be premature, and till now no successful analysis was made, even when examining specimens perfectly preserved in amber ([52]; for reviews see: [53,54,55]).

Even molecular research on recent taxa often do not provide us with sound basis for full phylogenetical research, as they do not represent extinct lineages. Therefore, fossil specimens are of fundamental value for such research, especially when they convey full information on the morphology of entire bodies, as the amber inclusions do.

The present discovery of new species in the beginning of Upper Cretaceous (Cenomanian) Burmese amber offers a new insight on taxonomy within Limoniidae. New materials enabled us a full reconstruction of male and female genitalia of a previously described species, *Drinosa prisca*. It was previously correctly observed that this species does not fit any known subfamily of limoniids [13]. The morphological features of newly discovered species *Decessia podenasi* gen. et sp. nov. inclined us to classify both species to a new subfamily and to designate a new genus.

**Figure 8:**
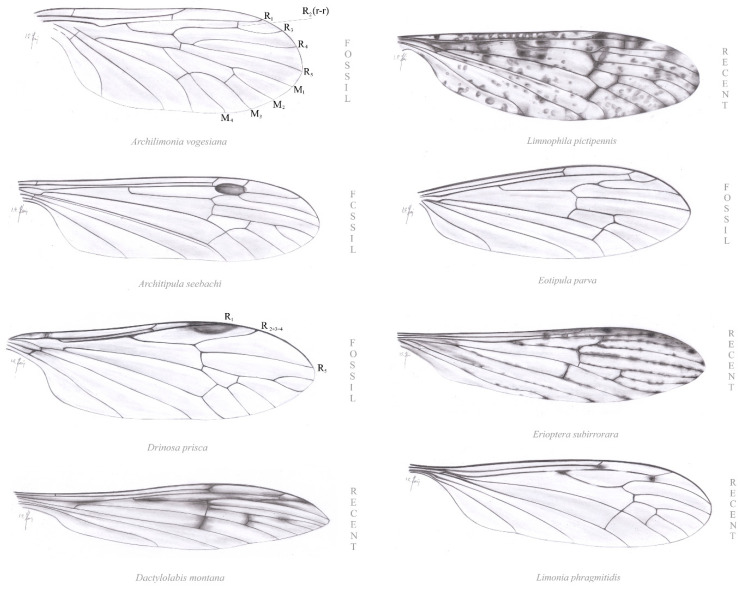
Wing venation of: Archilimoniidae: *Archilimonia vogesiana* Krzemiński et Krzemińska, 2003 [11], (redrawn after [11]). Limoniidae: *Architipula seebachi* Geinitz, 1884 [59], Architipulinae (redrawn after [49]); *Drinosa prisca* Podenas et Poinar, 2009 [13], Drinosinae subfam. nov. (redrawn after [13]); *Dactylolabis montana* Osten-Sacken, 1860 [19], Dactylolabinae *Limnophila pictipennis* Meigen, 1818 [18], Limnophilinae: *Eotipula parva* Handlirsch, 1906 [24], Eotipulinae (redrawn after [11]); *Erioptera subirrorata* Alexander, 1920 [22], Chioneinae; *Limonia phragmitidis* Schrank, 1781 [60], Limoniinae (redrawn after [61]).

Wing venation of the genus *Drinosa* was compared [13] with wing venation of representatives of the genera *Teucholabis* Osten-Sacken, 1859 [19] and *Gonomyia* in which a strong reduction of radial veins is visible (citation: “The wing venation of *Drinosa* resembles that of the genera *Teucholabis* and *Gonomyia* which are characterized by strongly reduced Rs branches with only two reaching the wing margin. The radial branches of *Drinosa* are even more reduced because of the lack of R_2_ vein with only R_3_ and R_4+5_ present” [13]: 471). In fact, in these genera the vein R_4+5_ does not exist, which is also true to all Limoniidae, save a few exceptions. In the Limoniidae, as well as in most Tipulomorpha, the vein Rs branches into veins R_2+3+4_ and R_5_ ([27,38]: 145). The presence of the vein R_4+5_ is characteristic only for the oldest evolutionary line of Tipulomorpha, the Archilimoniidae from the Middle Triassic [12,31], and to a majority of the Pediciidae, whose oldest fossil representatives are known from the Middle Jurassic ([25]: 56; [56]: 136; [57]: 240). Wing venation of representatives of all seven subfamilies of Limoniidae, and of the Archilimoniidae is shown in Figure 8. In the Limoniidae an exceptional, rare appearance of a very short section of vein R_4+5_ occurs, for instance, in the genus *Mesotipula* (subfamily Architipulinae); among several fossil species of this genus there is only one, *Mesotipula* (*Irenatipula*) *sigmoidea* Lukashevich, 2009 [56], with a very short section of vein R_4+5_ which seems to be an example of convergence in plesiomorphic furcation of the radial sector ([58]: Figure 3B).

In *Gonomyia* (subfamily Chioneinae) three branches of Rs reach wing margin, R_3_, R_4_, and R_5_; r-r is absent, probably fused with R_3_ ([36]: Figure 2; therein also extensive discussion on reduction in radial field in this genus).

## 5. Conclusions

The Drinosinae subfam. nov. shows a unique venation of wing, in which the extreme reduction of radial veins is combined with a complete set of four medial veins. This venation pattern results in an amazing appearance of the wing: the medial field is more densely set with veins than the radial field. The remaining six subfamilies of the Limoniidae show either the reverse combination, or both fields are more or less equally armoured with veins (Figure 8). The weak support of the radial field with veins in Drinosinae may indicate some unknown properties of the biomechanics of their flight.

The two genera, *Drinosa* and *Decesia*, that have exceptional wing venation deserve placement into a new subfamily, Drinosinae, and contribute to our understanding of the systematics and evolution of the Limoniidae. This particular venation is known only from one place and time: Cretaceous Burmese amber, and apparently did not survive to recent times.

## Figures and Tables

**Table 1:** A list of subfamilies of family Limoniidae, known till know (the age range follows that in the descriptions cited).

No.	Subfamily	Time Scale	The Oledest Known Representative/Species	Age Range (MA)
1.	Chioneinae Rondani, 1841 [21]	Cretaceous—Recent	*Gonomyia* (*Azaria*) *libanensis* Kania, Krzemiński, Krzemińska, 2015 [36]	140
2.	Dactylolabinae Alexander, 1920 [22]	Eocene—Recent	*Dactylolabis* (*Eobothrophorus*) *hoffeinsorum* Krzemiński, Kania, Krzemińska, 2010 [37]	45
3.	Limnophilinae Bigot, 1854 [20]	Cretaceous—Recent	*Austrolimnophila joana* Podenas, Poinar, 2009 [13]	98.79 ± 0.62
4.	Limoniinae Speiser, 1909 [23]	Cretaceous—Recent	*Helius ewa* Krzemiński, Kania, Azar, 2014 [38]	140
5.	Eotipulinae Kalugina, 1985 [25]	Jurassic	*Eotipula parva* Krzemiński, 1992 [27]	221.5–205.6
6.	Architipulinae Handlirsch, 1906 [33]	Triassic	*Architipula youngi* Handlirsh, 1906 [33]	183.0–182.0

## Data Availability

Data is contained within the article.
